# Effects of Climate Temperature and Water Stress on Plant Growth and Accumulation of Antioxidant Compounds in Sweet Basil (*Ocimum basilicum* L.) Leafy Vegetable

**DOI:** 10.1155/2020/3808909

**Published:** 2020-02-27

**Authors:** Asma Al-Huqail, Rehab M. El-Dakak, Marwa Nme Sanad, Reem H. Badr, Mohamed M. Ibrahim, Dina Soliman, Faheema Khan

**Affiliations:** ^1^Chair of Climate Change, Environmental Development and Vegetation Cover, Department of Botany and Microbiology, College of Science, King Saud University, Riyadh 11495, Saudi Arabia; ^2^Botany and Microbiology Department, Faculty of Science, Alexandria University, P.O. Box, 21511 Alexandria, Egypt; ^3^Genetics and Cytology Department, National Research Centre, Dokki-Giza, Egypt; ^4^Biology and Horticulture Department, Bergen College, Paramus, NJ 07652, USA; ^5^Kean University, College of Natural, Applied and Health Sciences, Biology Department, Union City, NJ 07083, USA

## Abstract

The effects of climate temperature and water stress on growth and several stress markers were investigated in sweet basil plants. Some growth parameters (shoot length and number of leaves) and photosynthetic chlorophyll contents were determined every two days during plant growth, and foliage leaf material was collected after 15 and 21 days of treatment. Both climate temperature and water stress inhibited sweet basil plant growth; especially, total chlorophyll levels were decreased significantly in response to high-temperature treatments. Under strong stresses, basil plants induced the synthesis and accumulation of glycine betaine (GB) as a secondary osmolyte, although at less content when compared with the proline content under the same stress conditions. Proline concentrations particularly increased in leaves of both basil stressed plants, accomplishing levels high enough to play a crucial role in cellular osmoregulation adjustment. Stress-induced accumulation of these antioxidant compounds was detected in sweet basil. Therefore, it appears that sweet basil-treated plants are able to synthesize antioxidant compounds under strong stress conditions. On the other hand, total sugar concentrations decreased in stress-treated basil plants. Both temperature and water stress treatments caused oxidative stress in the treated plants, as indicated by a significant increment in malondialdehyde (MDA) concentrations. An increase in total phenolic and flavonoid concentrations in response to water stress and a highly significant decrease in carotenoid concentrations in basil leaves were observed; flavonoids also increased under high climate temperature conditions.

## 1. Introduction

Plants are challenged by hostile environments of temperature, drought, salinity, and heavy metals which disrupt cellular and developmental processes. In arid or semiarid zones, high temperature and water stress are common climate factors that can significantly affect and diminish crop yields. Plants have developed complex and well-organized mechanisms to adopt and tolerate biotic and abiotic stress conditions [1]. Abiotic stresses such as water stress, salinity, cold temperature, anoxia, high light intensity, and nutrient imbalances usually and highly significantly affect plant growth, development, and their productivity. Plants accumulate many small organic molecules called osmolytes to protect their cellular machinery against environmental stresses [2–4]. Thus, it is important to understand the various mechanisms controlling different metabolic pathways and their interplay during different abiotic stresses and develop improved crop varieties for realizing complete yield potential. To combat stress, plant metabolism is altered in many different ways, including redox metabolism to remove excess levels of active oxygen species (AOS) and re-establish the cellular redox balance, and compatible solute production to stabilize proteins and cellular structures and/or to maintain cell turgor by osmotic adjustment [5–7]. The synthesis and accumulation of osmoprotectants (“osmolytes”) in the cytoplasm, a conserved phenomenon observed in all plants, including those tolerant and sensitive to stress, is considered as one of the most general responses to all abiotic stress conditions [8, 9]. Osmotic adjustment has been shown to be an effective component of stress tolerance, and accumulation of osmoprotectants such as proline, glycine betaine, gamma amino butyric acid (GABA), and sugars is a common molecular response observed in different plant compartments [3, 10]. Osmoprotectants named “osmolytes” are exceedingly compatible solutes, with a noticeable low molecular weight and highly soluble organic compounds that do not interfere with consistent and normal metabolism even when present at high concentrations. It has been demonstrated that some stress conditions such as temperature, water stress, salinity, and flooding, in general, resulted in increased soluble sugar levels, whereas low sugar levels are knowingly and perceptively seen under other abiotic stress conditions such as high light intensity irradiance, excess heavy metals, and nutrient deficiency [11]. Accumulation of different osmoprotectants results in an advantage for stressed plants by reducing the osmotic potential to maintain their turgor pressure even at low water potential resulting from stress. Persistence of the turgidity is very essential for cell expansion in addition to various biochemical pathways and processes such as photosynthesis and maintaining enzymatic activities [3].

Moreover, osmoprotectant “osmolytes” could also serve as a stabilizer for the cell membrane and proteins under water stress conditions and also act as protectants for cells against oxidative stress by deactivating active oxygen species {AOS} [12]. Proline and other common compatible osmolytes such as glycine betaine could probably be the most commonly formed osmoprotectant inside many plant compartments in response to different abiotic stresses [4]. The multiple functions and roles of sugars as essential molecules in several primary metabolic and regulatory pathways can mask and interrupt their role as active osmolytes in different plant species and under various kinds of stresses [13]. Most of the abiotic stresses, including high temperature and water stress, result in the synthesis of several active oxygen species (AOS), including singlet oxygen (^1^O_2_), hydroxyl radical (OH), superoxide anion radical (O_2_^−^), hydrogen peroxide (H_2_O_2_), and other highly active oxidant molecules [11]. Consequently, continuous increment of the generated AOS leads to the existence of oxidative stress, which rapidly damages proteins, DNA, cellular membranes, and several other macromolecules [14].

In response to different abiotic and biotic stresses, plants are equipped with complex and highly efficient antioxidative defense systems composed of protective nonenzymatic antioxidants such as ascorbic acid (vitamin C), *α*-tocopherol (vitamin E), carotenoids, flavonoids, and phenolic compounds and enzymatic antioxidants such as superoxide dismutase (SOD, EC 1.15.1.1), catalase (CAT, EC 1.11.1.6), and ascorbic peroxidases (APX, EC 1.11.1.11) that act to maintain the reduced form of the antioxidants and subsequently scavenge the active oxygen species [15, 16]. One of the most effective nonenzymatic antioxidant defense metabolites is carotenoids which perform several indirect protective functions, including their function as an essential nonenzymatic antioxidant metabolite in stressed plants, besides their direct role as pigments in photosynthesis [11]. Furthermore, polyphenols including flavonoids are considered the most complex subgroup, including many thousands of different bioactive compounds with a wide range of unique metabolic functions in all plants [17]. These phenolic compounds are usually characterized as common important cell wall components of plant cells, and they are highly involved in the defense mechanisms toward most of the abiotic stresses, particularly UV-irradiation, water stress, high temperature, heavy metals, and salinity, among others [18, 19].

Responses of basil to abiotic stresses, within its tolerance range under natural conditions, have been extensively studied; however, the physiological response and biochemical processes of basil to extremely higher temperature and water stress need more research. Plants exposed to these extreme heat shock-induced abiotic stress treatments, higher than the heat they are exposed to under normal field conditions, may activate certain adaptive and protective mechanisms that cannot be observed under low or moderate level of those stresses [20]. These two abiotic stresses, which are considered to be a consequence of recent climatic changes, could significantly lessen the production of many crops and vegetables in different countries in the coming few years. The present study aims to investigate the effect of high climate temperature and water stress on the growth of basil plants; these stresses were applied under various conditions, including high-temperature treatments beyond the tolerance threshold, to give us the opportunity to detect the exposure duration- and temperature-dependent effects. According to the regular and common mechanisms in stressed plants, in the present study we quantified and measured the growth responses accompanied with the accumulation of certain stress biomarkers: osmoprotectants (total sugars, proline, and glycine betaine), responsible for cellular osmotic adjustment; malondialdehyde (MDA), as a marker of lipid peroxidation of plant tissues that results from oxidative stress; and some antioxidant nonenzymatic metabolites (carotenoids, total phenolics, and several flavonoids), synthesized as a secondary response to oxidative stress. The experiments were carried out in basil, a variety that has a growing market [17, 19].

## 2. Materials and Methods

### 2.1. Plant Material

Sweet basil (*Ocimum basilicum* L.) is the most popular herb grown globally and is also sometimes called “common basil.” Seeds of sweet basil were provided by Amazon from Eden brothers.com (Brevard Road, Arden, NC, USA).

### 2.2. Plant Growth

Seeds of sweet basil (*Ocimum basilicum* L.) were surface-sterilized by immersion for 2 min in 0.1% HgCl_2_; thereafter they were washed with five changes of sterile distilled water. The seeds were soaked in continuously aerated distilled water for 12 h in darkness. At the end of the soaking period, the seeds were sown in plastic seed trays filled with washed pure quartz sand. All pots were placed in a growth chamber under 75–80% relative humidity under a long-day photoperiod (16 h light/8 h dark) with regulated temperatures ranging between 18 and 25°C. Light intensity was 420 *μ*mol m^−2^ s^−1^ at the top of plants, supplied by a mixture of fluorescent and incandescent lamps. Young plants were transferred to individual plastic pots (15 cm diameter × 20 cm height) filled with prewashed pure quartz sand on the same substrate 35 days after sowing and were watered for 15 additional days with a standard nutritive Hoagland solution. Then, temperature and water stress treatments were started, by warming the plants with increasing temperatures (35, 45, and 55°C) and by stopping irrigation altogether, respectively; 12 plants were used per treatment. Irrigation was carried out twice a week by adding 1.5 L of the nutritive solution to each tray, which contained 12 standard pots. Control plants were grown in parallel, maintaining the standard irrigation regime with the nutritive solution. After starting the treatments, plant height including shoot and root length was measured, and the number of leaves per plant was counted at regular intervals every two days, to assess the effect of stress on vegetative plant growth parts. For each stress treatment, including nontreated controls, leaves at equivalent positions (the second leaves) were detached from all plants, 15 and 21 days after starting the temperature and water stress treatments. Just after harvest, the whole plants or dissected organs were blotted dry and weighed carefully on a precision balance to calculate the average fresh weight related to each treatment, and then dried in a hot-air oven at 65°C until a constant weight to obtain the dry weight measure for each treatment. Also, the percentage water content was calculated for each treatment according to the following equation: [(FW − DW)/FW] × 100. For biochemical analyses, the leaf material was stored at −80°C until further use. Photosynthetic pigments, *viz*, chlorophyll *a*, chlorophyll *b*, and total carotenoids, were also determined at the same time, following the procedures described by Sims and Gamon [21]: 0.2 g fresh leaf was crushed in 80% acetone/Tris buffer (ice cold) for 1 h and then centrifuged for 15 min at 3,000 ×g. The supernatant was used to measure the optical density at 663 nm, 647 nm, and 470 nm. Total carotenoid values were then converted into *μ*g·g^−1^ DW. The formulae used for the determination of photosynthetic pigments were as follows:

(1) $$\begin{array}{c} \text{Chl }a=10.3E_{663}-E_{647},\\ \text{Chl }b=19.7E_{647}-E_{663},\\ \text{Carot}=4.2E\;470-\left(0.0264\text{ Chl }a+0.426\text{ Chl b}\right). \end{array}$$

### 2.3. Electrolyte Leakage

Electrolyte leakage was measured according to the method described by Di-Cagno et al. [22]. Leaf pieces (1 cm) were collected and washed with deionized water to remove surface-adhered electrolytes and incubated overnight at 25°C, with gentle shaking on a gyratory shaker. After measurements using a conductivity meter (4026 data, Haniz Instruments, Padova, Italy), the tubes were then placed in boiling water for 10 min and then cooled to room temperature. Conductivity was again determined. Electrolyte leakage was calculated as the ratio of conductivity before and after boiling.

### 2.4. Determination of Osmolytes (Proline, Glycine Betaine, and Total Sugar)

Proline contents (Pro) were determined following the procedures described by Bates et al. [23] with minor modification [24]. Frozen leaf segments (150 mg) were ground in a mortar into fine powder in 3% aqueous sulphosalicylic acid and homogenized. The homogenate was centrifuged at 12,000 g for 10 min. One volume of the filtrate was added to one volume of the prepared ninhydrin solution. The reaction mixture containing 100 mL volume of the supernatant, 100 mL glacial acetic acid, and 100 mL ninhydrin reagent (2.5% ninhydrin in 60% phosphoric acid) was incubated at 98°C for 30 min and terminated by cooling the tubes on ice. Absorbance was determined at 518 nm. After that, the samples on ice were extracted with two volumes of toluene, which was used as a blank. Proline concentrations in the plant leaves were expressed as “*μ*mol proline/g DW.”

The extraction and quantification of glycine betaine (GB) was determined according to the procedures of Grieve and Grattan [25] with some additional modifications recommended by Nawaz and Ashraf [26]. Frozen plant leaves (100 mg) were homogenized in a volume of 3 mL dd-water and centrifuged at 13,000 rpm at −4°C for 10 minutes. After that, 1 mL from the supernatant was mixed with 400 *μ*L 2 N HCl. After stirring, 200 *μ*L from each tube was mixed with 80 *μ*L of KI and brought to 100 mL volume with dd-water. The tubes were quickly placed in ice and stirred for 20 min. After one and half hours, 600 *μ*L of cold dd-water and 3 mL of dichloroethane were added and the mixture kept at −20°C until used. After the samples were vortexed for 40 seconds and then allowed to settle until the organic phases were completely separated; at the end, the absorbance of 1 mL of the lower aqueous phase was measured at 265 nm using a UV spectrophotometer. A standard curve was obtained, using glycine betaine (GB) of increasing concentrations in dd-water under the same conditions as the plant samples. Glycine betaine (GB) contents in the plant samples were expressed as “*μ*mol (GB)/g DW.”

According to the method described by Dubois et al. [27], total sugars were quantified in basil plant materials. Dried leaves (100 mg) were crushed in 3 mL of 75% methanol and then mixed with 5% phenol in concentrated H_2_SO_4_; finally, absorbance was measured at 490 nm in a visible spectrophotometer. Glucose was used as standard, and the amount of total soluble sugars in basil leaves was calculated as “mg. equ. G/g DW.”

### 2.5. Malondialdehyde and Antioxidants

The same extract used for the determination of total soluble sugars (0.1 g of dried plant leaves in 3 mL of 80% methanol) was also used for the quantification of malondialdehyde (MDA), total phenolic, and flavonoid concentrations.

Malondialdehyde (MDA) content was assayed as an excellent indicator of oxidative stress and is usually measured to assess the extent of lipid peroxidation in leaf tissues by the method of Del Rio et al. [28]. MDA concentration was determined by using thiobarbituric acid to be able to form stable complex thiobarbituric acid-reactive substances (TBARS). Absorbance was read at 600 nm. MDA concentration was calculated using a molar extinction coefficient of 155 mM^−1^ cm^−1^.

Total phenolic compounds were determined as reported by Sgherri et al. [29], extracted from fresh leaves (1 g) for 1 h with the Folin-Ciocalteu reagent and 50% methanol containing 1% HCl under continuous stirring at room temperature. After centrifugation at 12000 ×g for 15 min, the supernatant was collected and the extraction was repeated three times to obtain the pellet. Methanolic extracts were collected, dried, and resuspended in 80% methanol. A measure of total phenolic compounds (expressed in mg eq. gallic acid) was obtained by recording absorbance at 765 nm and using gallic acid as a standard. The calculations were performed using the absorbance and by using a calibration curve for total phenolics. Flavonoid concentrations were determined according to the method described by Mirecki and Teramura [30]. Flavonoids were extracted from fresh leaves grounded in 10 ml acidified methanol (79 : 20 : 1, v/v methanol/water/HCl). Absorbencies of centrifuged extracts were measured at 510 nm after appropriate dilution, and the amount of flavonoids was expressed in mg eq. Cat g^−1^ DW equivalents of catechin.

### 2.6. Statistical analysis

Data analysis was performed using the SPSS program by means of one-way ANOVA. The significance of differences between average values was obtained from samples. All estimates of sample variability are given at the 95% confidence level. The Tukey test was used to estimate homogeneous groups when more than two samples were compared.

## 3. Results

### 3.1. Electrolyte Leakage

**Figure 1:**
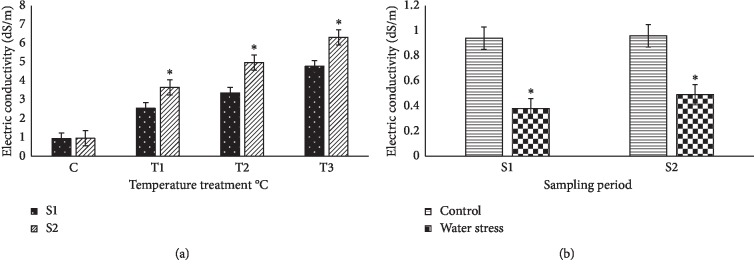
(a, b) Effect of different climate temperatures (C, 25°C; *T*_1_, 35°C; *T*_2_, 45°C; and *T*_3_, 55°C) and water stress (WS) treatments on the electric conductivity (dS/m), measured in soil:water (1 : 5) extracts after two sampling periods (S1, 14 days and S2, 21 days) in basil plants (mean ± SD, *n* = 5). Asterisks indicate significant differences between sampling periods of each treatment and differences between treatments for the same sampling periods according to the Tukey test (*α* = 0.05).

Electrolyte leakage of the plant leaves was determined before and after the climate temperature and water stress treatments (Figure 1). In the control nontreated plants, value at the end of the experiment was significantly higher than the initial one; this can be technically clarified by the accumulation of ions present in the Hoagland solution. Electrolyte leakage increased even more after treating the plants with different temperature treatments, in a temperature-dependent manner. The average electrolyte leakage also increased slightly in water-stressed plants, as compared with the initial value—most likely due to the accumulation of ions from the Hoagland solution—but the difference was not significant.

### 3.2. Stress Impact on Plant Growth

**Figure 2:**
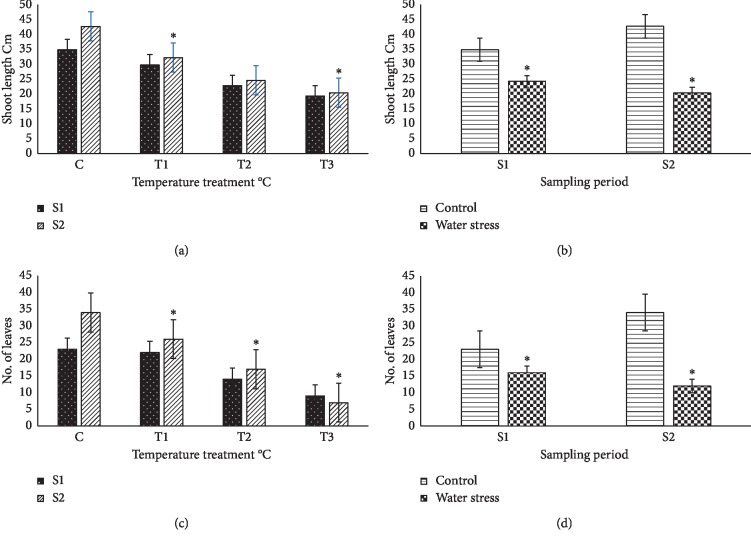
Effect of different climate temperatures (C, 25°C; *T*_1_, 35°C; *T*_2_, 45°C; and *T*_3_, 55°C) and water stress (WS) treatments on the shoot length (a, b) and number of leaves (c, d) after two sampling periods (S1, 14 days and S2, 21 days) in basil plants (mean ± SD, *n* = 10). Asterisks indicate significant differences between sampling periods of each treatment and differences between treatments for the same sampling period according to the Tukey test (*α* = 0.05).

High climate temperature and water stress induced significant inhibition of basil plant growth and negatively affected vegetative plant growth, which was measured by the increase in stem length and the number of leaves during the 21 days of treatment (Figure 2). As compared to the nontreated controls, all temperature treatments tested totally blocked the growth of sweet basil plants, with a clear temperature-dependent effect: the higher the temperature stress used, the shorter the lag period before growth inhibition was observed (Figures 2(a) and 2(c)). Water stress also inhibited stem growth and the increase in leaf number; this effect was clearly and significantly observed 10 days after the last irrigation with Hoagland nutritive solution, once the soil had dried (Figures 2(b) and 2(d)).

**Figure 3:**
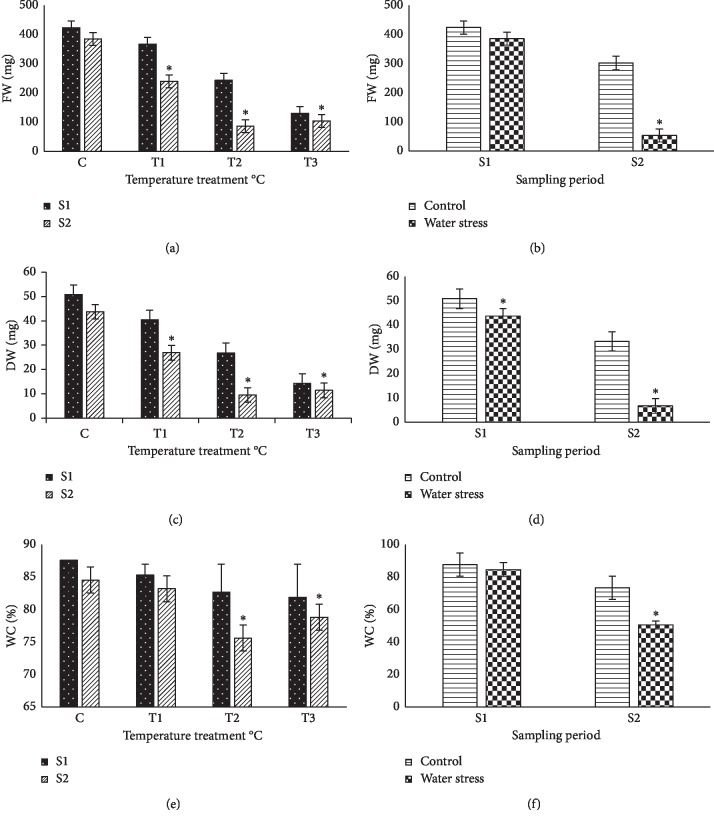
Effect of different climate temperatures (C, 25°C; *T*_1_, 35°C; *T*_2_, 45°C; and *T*_3_, 55°C) and water stress (WS) treatments on fresh weight, FW (a, b); dry weight, DW (c, d); and water content (e, f) after two sampling periods (S1, 14 days and S2, 21 days) in basil plants (mean ± SD, *n* = 10). Asterisks indicate significant differences between sampling periods of each treatment and differences between treatments for the same sampling period according to the Tukey test (*α* = 0.05).

Apart from the standard growth parameters measured continuously, stress-induced inhibition of plant growth was also assessed by determining the mean fresh weight (FW) and dry weight (DW) of the basil leaves after 15 days (sampling 1) and 21 days (sampling 2) of temperature and water stress treatments (Figure 3). A significant high climate temperature-dependent decrease of fresh weight was observed in temperature-treated basil plants, reaching approximately 73% reduction in those exposed to 55°C, with respect to the nontreated controls. Under this high temperature, the plants were already strongly affected after 21 days of treatment, and no significant differences were found between the two samplings; at the other two temperatures, −35 or 45°C, reduction of FW increased with the period of treatment duration (Figure 3(a)). Similar qualitative results were also obtained when the basil plants were submitted and exposed to water stress, although with stronger differences between the two samplings: after 15 days without irrigation, plant FW was reduced by nearly 25%, on average, but by almost 89% after 21 days of treatment (Figure 3(b)). The relative decrease in FW under different water stress conditions—as compared with the control plants—was not only a consequence of the inhibition of growth, but also due to a significant loss of water, as shown by the calculated water content of the sweet basil leaves: it was only 50% of the FW for the plants maintained for 21 days without water, while for those normally irrigated with nutritive solution, the water content was about 85% of the total fresh leaf weight of basil plants (Figure 3(d)). Under various climate temperature stresses, a general, but not statistically significant, decrease of the mean leaf water content was observed with increasing temperature, both after 14 and 21 days of treatment, whereas significant differences were markedly detected between the samples collected at different times for each temperature treatment, up to 55°C (Figure 3(c)).

### 3.3. Chlorophyll Content and Total Carotenoids

**Figure 4:**
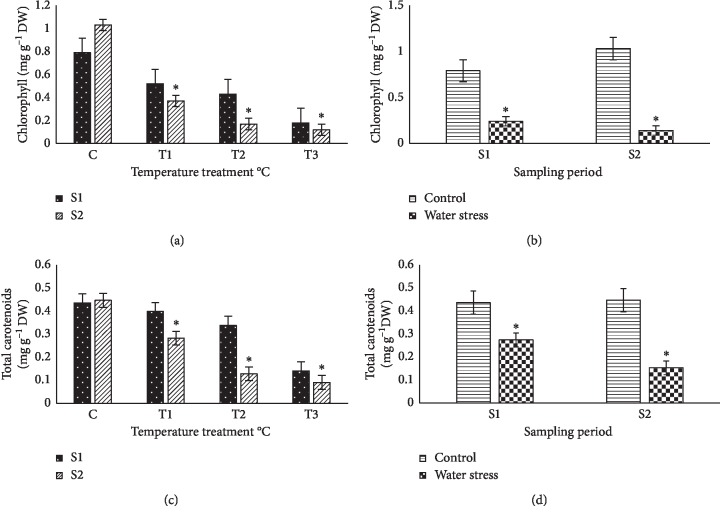
Effect of different climate temperatures (C, 25°C; *T*_1_, 35°C; *T*_2_, 45°C; and *T*_3_, 55°C) and water stress (WS) treatments on total chlorophyll (a, b) and total carotenoids (c, d) after two sampling periods (S1, 14 days and S2, 21 days) in basil plants (mean ± SD, *n* = 10). Asterisks indicate significant differences between sampling periods of each treatment and differences between treatments for the same sampling period according to the Tukey test (*α* = 0.05).

The chlorophyll content of crop plants is positively correlated with their photosynthetic activity [31], and a reduction of chlorophyll level contributes to the inhibition of photosynthesis observed under various abiotic stress conditions. A high decrease in chlorophyll levels in comparison with the nontreated control plants was indeed observed in plants exposed to high temperature (45 and 55°C) for two weeks or longer periods (Figure 4(a)). On the other hand, no significant differences with nonstressed control basil plants were observed when a moderate temperature (35°C) was used (Figure 4(a)) or when the basil plants were subjected to the water stress treatment (Figure 4(b)). Total carotenoid levels in leaves of basil plants decreased in all treatments in comparison with nontreated control plants. In plants treated with the highest temperature (55°C), this reduction was about 50%. When comparing the two sampling periods, lower values were found in plants exposed for a prolonged time to stress in all treatments (Figures 4(c) and 4(d)).

### 3.4. Glycine Betaine

**Figure 5:**
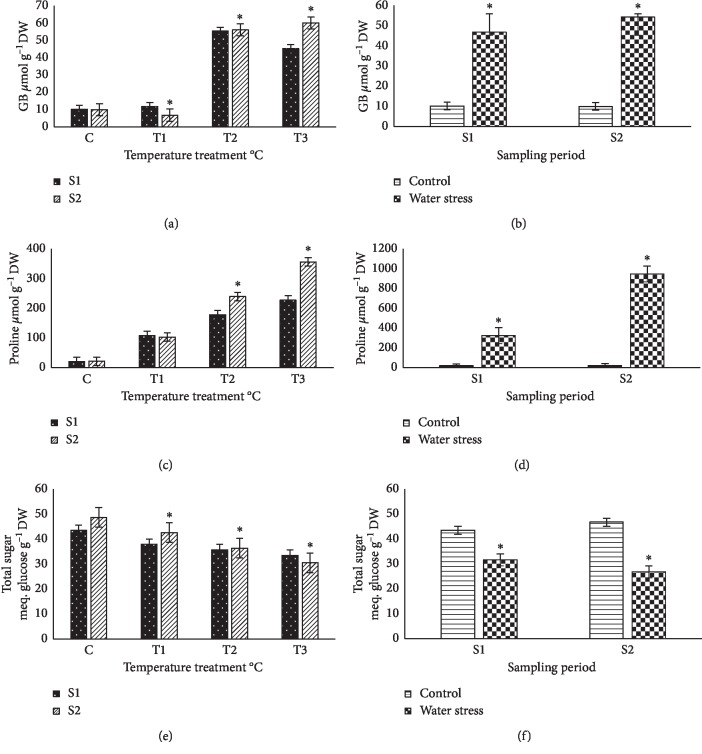
Effect of different climate temperatures (C, 25°C; *T*_1_, 35°C; *T*_2_, 45°C; and *T*_3_, 55°C) and water stress (WS) treatments on glycine betaine (GB) (a, b), proline (c, d), and total sugar contents after two sampling periods (S1, 14 days and S2, 21 days) in basil plants (mean ± SD, *n* = 10). Asterisks indicate significant differences between sampling periods of each treatment and differences between treatments for the same sampling period according to the Tukey test (*α* = 0.05).

High-temperature and severe water stress treatments also activated the synthesis and accumulation of glycine betaine (GB) in basil plants (Figures 5(a) and 5(b)). The total glycine betaine (GB) content reached 50–60 *μ*mol per gram DW in plants exposed to 45^◦^C and 55^◦^C in comparison with nontreated control plants (=∼5-fold), which were less than those of proline. Moreover, no time-dependent increase in glycine betaine (GB) levels was observed in our experiment, as the values quantified were not significantly different in two sampling plant materials (Figure 5(a)). Similar results were obtained in water stress plants after 14 or 21 days of treatment (Figure 5(b)).

### 3.5. Proline Content

Exposure to various climate temperatures significantly increased proline levels in sweet basil plant leaves, in a temperature-dependent tendency. The average values of proline under 45 and 55°C external climate temperature also increased with prolonged time of experimental treatment, although the differences observed between the two samplings of plant material were significant only at 55°C. Under the highest temperature conditions tested, proline levels reached about 356.4 *μ*mol g^−1^ DW, which represented a significantly high increment, which was 15-fold higher than that of nontreated control basil plants (Figure 5(c)). Proline accumulation was more significantly increased and clearly observed in plants under water stress conditions, with increases of about 20-fold and 36-fold after 14 and 21 days, respectively, of water stress (Figure 5(d)). Proline concentrations in the plants subjected to the longest water stress treatment (21 days without irrigation), in terms of dry weight (946.8 *μ*mol g^−1^), were about double that of those determined in plants treated for the same time with the highest temperature (Figures 5(c) and 5(d)); however, considering the drastic reduction in water content in nonirrigated plants, it can be said that both treatments induced proline accumulation to slightly parallel levels, in absolute terms.

### 3.6. Total Soluble Sugars

The total amount of soluble sugars slightly decreased in temperature-stressed plants, but significant differences were detected only starting with 45°C in both samplings (Figure 5(e)). Under water stress conditions, the decrease was also significant and highly detected after a longer water stress treatment (Figure 5(f)).

### 3.7. Malondialdehyde (MDA)

Malondialdehyde (MDA) is considered a reliable marker of oxidative stress and is a product of membrane lipid peroxidation; thus, higher MDA contents should correspond to a higher degree of oxidative stress. MDA concentrations increased with all treatments, mostly after longer exposure to stress, so higher values were determined in the second sampling, after 21 days of treatment (Table 1). The treated plants most affected by oxidative stress were those treated with 55°C, in which the highest MDA contents were measured (Table 1).

### 3.8. Total Phenolics and Flavonoids

Contrary to the trend of variation of total carotenoids, total phenolics increased in plants under stress, especially in those basil plants treated with high temperature. Values were significantly higher in plants from the second sampling than in those from the first one in all stress treatments (Table 1). A different response to the type of stress was observed in total flavonoid contents, which increased highly significantly in response to temperature stress but decreased under water stress conditions (Table 1).

### 3.9. Discussion

Crops and wild species are affected relatively by environmental stress conditions such as temperature, water stress, or high salinity, but basil plants prefer moderately high soil and climate temperature; therefore, they can be cultivated in regions exposed to a certain degree of high temperature [32]. Yet, high soil temperature inhibits seed germination and plant growth, and causes reduction in crop yields [1]. In this study, young sweet basil plants were grown with increasing climate temperature, and temperature- and time-dependent inhibition of vegetative growth has been observed, which was better shown by the reduction of fresh weight of the plants; this was not unexpected since inhibition of growth is probably the most general response of plants to stress [1]. In the experimental conditions used here, the water content of the plants was reduced very slightly in response to different climate temperature treatments, suggesting that basil plants activate relatively efficient mechanisms to cope with temperature stress and that growth inhibition is mostly due to the effect of high temperature on plant metabolism. Water stress also inhibited growth but, contrary to temperature, in this case severe dehydration of the plants was observed. Another difference between temperature and water stress treatments refers to changes in chlorophyll contents. In plants treated with high temperatures, a significant reduction in chlorophyll levels, up to ca. 40% of that in the nontreated control plants, was observed. The decrease in chlorophyll levels in plants affected by temperature is due to the inhibition of chlorophyll synthesis, together with the activation of its degradation by the enzyme chlorophyllase [33]. Although water stress should also reduce plant chlorophyll levels and photosynthetic activity [34], this was not observed in the present study probably because the time that the plants were maintained without irrigation was not long enough to detect these effects. Proline is generally considered as a good indicator of environmental stress in different pants including basil [35], and there are many reports describing an increase in proline contents as a response to water and temperature stress in this species [20, 36, 36], although it should be mentioned that data specific for basil are rather scarce [14]. What is not so clear is the possible contribution of significant accumulation of osmolyte proline to the relative resistance of basil plants to high climate temperature. When comparing basil cultivars with differences in their degree of high temperature tolerance, in some cases the more tolerant cultivars were found to synthesize higher amounts of proline under treatment stress [36], but in others there was no correlation between tolerance and proline levels [35]. In the present study, a clear positive correlation between proline accumulation and the intensity of the applied stress treatments has been established, that is, of proline levels with the electric conductivity of the substrate—reflecting increasing climate temperature—and with the time of exposure to stress. Moreover, osmolyte proline content reached marked levels that were high enough to play a significant role in cellular adjustment under these stress conditions. Taken together, these data strongly support the notion that proline is the major physiological osmolyte in basil, as it has been suggested for other basil varieties [35]. Usually, a given different plant species accumulates preferentially a particular type of osmolyte in response to environmental stress; there have even been attempts to use this preference for one specific type of compatible solute as a taxonomic criterion in wild species [37]. In agreement with this idea, and since proline appears to be the major osmolyte in basil, it is generally accepted that basil does not accumulate glycine betaine in natural conditions; in fact, it has been reported that glycine betaine N,N,N‐trimethylglycine is the widely accumulated osmolyte in plants and other organisms [10]. In the case of osmoregulation, the compatible solute glycine betaine (GB), a small organic metabolite, can potentially play a crucial role in effective protection against high salinity, drought, and extreme temperature stress [10, 38]. Conversely, many plants under severe stress treatments showed significant activity in metabolic pathways that form osmoprotectants to help ameliorate the destructive effect of stress, by using these osmolytes for osmotic adjustments and/or acting as osmoprotectants, as it has been recently observed and discussed [13, 37]. According to our results, this seems to be the status for basil: contrary to the general assumption, in our study we detected a marked accumulation of glycine betaine (GB) in basil plants under high-temperature and water stress treatments. Indeed, the highest level of glycine betaine (GB) measured (=∼ 50 *μ*mol g^−1^ DW) was much more lower than the amount of proline in the same plant under the same treatments (about 10-fold less) and much lower than other taxa that are glycine betaine (GB) accumulators [13, 39]. Therefore, glycine betaine (GB) will have only an indirect effect on osmotic balance in basil stressed plants, but may persist in participating significantly to help in stress resistance due to its putative functions as an AOS scavenger and/or chaperon. Our results evidenced the marked accumulation of glycine betaine (GB) in basil plants under stressed conditions, and these results are consistent with the results obtained by Ekren et al. under different water level treatments [35]. The increase in sugars after mild temperature and water stress is well known in basil leaves, and moderate abiotic stress was even recommended as a strategy for improving the quality of certain plants [40]. Temperature stress was found to produce an increase in total carbohydrate accumulation [41]. There are also several reports of increased amount of sugars in basil under drought [42]. However, there are relatively few data published on stress-induced sugar accumulation in other vegetative organs in this species, and they generally indicate a reduction in total carbohydrates in leaves [43, 44], as it has been found in the present study. In addition to sugars, basil leaves are rich in several compounds considered as “health-promoting,” such as carotenoids, flavonoids, and other phenolics. These secondary metabolites play multiple roles in plants, including scavenging of active oxygen species (AOS) induced under different stress conditions and causing oxidative stress. A clear symptom of oxidative damage is cell membrane degradation; therefore, MDA—a product of membrane lipid peroxidation—is an excellent marker of oxidative stress [28]. In this study, a significant increase in MDA levels in basil leaves upon temperature and water stress treatments of the plants was observed, in agreement with previous reports showing a temperature-induced increase in MDA contents in basil leaves [45]. Mild and moderate temperature and water stress also produce an increase in carotenoid levels in basil leaves. Carotenoid is known as an important natural antioxidant with anti-carcinogenic properties [46]. In the present work, carotenoid levels were measured in basil leaves and a reduction was detected under stress conditions; this is in agreement with previous reports that found a negative correlation between temperature and carotenoid contents in basil leaves [47]. Temperature stress led to a significant increase in total phenolics and flavonoids in leaves of plants subjected to the temperature treatment and an enhancement of the former in water-stressed plants. There are many publications reporting an increase in the levels of phenolics and flavonoids in basil plants in conditions of abiotic stress, which is a topic of direct interest for human health [48, 49]; in fact, consumption of basil leaves has been recommended to reduce the risk of many diseases [42]. Similar studies on leaf material are much scarcer; for example, Nguyen et al. [50] found an enhancement of some phenolics and flavonoids under moderate water stress (50% of the field capacity) in the more tolerant cultivars of basil plants, but a reduction in the more sensitive ones, in partial agreement with the data presented here.

## 4. Conclusions

Temperature and water stress treatments inhibited vegetative growth in basil plants, a variety that has not been extensively studied yet, despite its growing commercial interest. In nonirrigated plants, strong dehydration was partly responsible for the reduction of leaf fresh weight, an effect not detected in response to high-temperature treatment. Both stresses led to the accumulation in the leaves of high levels of proline, which functions as the major osmolyte in basil, responsible for osmotic adjustment under stress conditions. Glycine betaine (GB) also accumulated as a response to temperature and water stress—although at marked lower levels than proline; therefore, glycine betaine (GB) acts as an indirect secondary osmoprotectant compound that could contribute to osmolyte tolerance toward different abiotic stresses in basil plants. Both stress treatments caused secondary oxidative stress in basil plants, as indicated by the significant increase in malondialdehyde (MDA) contents and hydrogen peroxide (H_2_O_2_). The increase in antioxidant total phenolic compound levels in leaves can be considered as part of the response induced to cope with oxidative stress. Contrary to what has been reported for basil leaves, other metabolites such as total soluble sugars or carotenoids do not increase but rather decrease in leaves in response to the stress treatments.

## Figures and Tables

**Table 1:** Effect of different climate temperatures (C, 25°C; *T*_1_, 35°C; *T*_2_, 45°C; and *T*_3_, 55°C) and water stress (WS) treatments on malondialdehyde (MDA), total phenolics, and total flavonoids after two sampling periods (S1, 14 days and S2, 21 days) in basil plants (mean ± SD, *n* = 10). Different lower case letters indicate significance differences between treatments for the same sampling period according to the Tukey test (*α* = 0.05).

Parameter	Sampling periods	Stress treatments					
		Temperature stress (°C)				Water stress	
		C	*T*_1_	*T*_2_	*T*_3_	C	WS
Malondialdehyde (MDA), nmol g^−1^ DW	S1	143.2 ± 10.10**a**	161.6 ± 9.63**ab**	169.9 ± 8.21**ab**	215.2 ± 7.31**ab**	143.2 ± 10.10**a**	172.8 ± 8.65**b**
	S2	147.4 ± 9.67**a**	187.4 ± 8.97**b**	204.7 ± 7.29**bc**	236.6 ± 6.32**c**	147.4 ± 9.67**a**	197.8 ± 6.88**b**
Total phenolics, mg eq “GA” g^−1^ DW	S1	9.72 ± 0.16**a**	11.46 ± 1.58**ab**	12.97 ± 1.64**b**	14.98 ± 0.82**c**	9.72 ± 0.16**a**	12.31 ± 1.89**b**
	S2	12.39 ± 0.63**a**	17.97 ± 0.36**b**	15.58 ± 3.30**b**	17.86 ± 2.31**b**	12.39 ± 0.63**a**	19.38 ± 0.91**b**
Total flavonoids, mg eq “C” g^−1^ DW	S1	10.22 ± 0.32**a**	11.13 ± 0.82**b**	12.22 ± 0.83**b**	12.91 ± 0.52**b**	10.22 ± 0.32**a**	7.25 ± 0.23**d**
	S2	8.72 ± 0.59**a**	11.23 ± 1.00**b**	11.6 ± 0.53**b**	13.69 ± 0.54**c**	8.72 ± 0.59**a**	5.13 ± 0.54**d**

## Data Availability

The data used to support the findings of this study are included within the article.
