# Corrigendum to “Effects of Climate Temperature and Water Stress on Plant Growth and Accumulation of Antioxidant Compounds in Sweet Basil (*Ocimum basilicum* L.) Leafy Vegetable”

**DOI:** 10.1155/2020/9362517

**Published:** 2020-11-27

**Authors:** Asma Al-Huqail, Rehab M. El-Dakak, Marwa N. M. E. Sanad, Reem H. Badr, Mohamed M. Ibrahim, Dina Soliman, Faheema Khan

**Affiliations:** ^1^Chair of Climate Change, Environmental Development and Vegetation Cover, Department of Botany and Microbiology, College of Science, King Saud University, Riyadh 11495, Saudi Arabia; ^2^Botany and Microbiology Department, Faculty of Science, Alexandria University, P.O. Box 21511, Alexandria, Egypt; ^3^Genetics and Cytology Department, National Research Centre, Dokki-Giza, Egypt; ^4^Biology and Horticulture Department, Bergen College, Paramus, NJ 07652, USA; ^5^Kean University, College of Natural, Applied and Health Sciences, Biology Department, Union City, NJ 07083, USA

In the article titled “Effects of Climate Temperature and Water Stress on Plant Growth and Accumulation of Antioxidant Compounds in Sweet Basil (*Ocimum basilicum* L.) Leafy Vegetable” [1], there was a spelling error in author Marwa Sanad's name in the author list, where Marwa Nme Sanad should have read Marwa N. M. E. Sanad. This is corrected as shown above.
